# Effect of ACDF combined with different degrees of partial resection of uncovertebral joints on cervical stability and degeneration: a three-dimensional finite element analysis

**DOI:** 10.1186/s13018-022-03447-0

**Published:** 2022-12-19

**Authors:** Wei Guo, Yuan Jiang, Yang Zhu, Jingwen Huang

**Affiliations:** Department of Spine Surgery, The First People’s Hospital of Changde City, No. 818, Renmin Road, Changde, 415000 Hunan Province People’s Republic of China

**Keywords:** Cervical spine radiculopathy, Uncovertebral joints, Cervical stability, Finite element analysis

## Abstract

**Background:**

To evaluate the influence of the resection of different amounts of the uncovertebral joints on the stability of the cervical spine by comparing and analyzing the stress distribution and peak displacement characteristics of the internal fixation structures and endplates.

**Methods:**

After obtaining the CT data of a 34-year-old male healthy cervical spine, a three-dimensional finite element model was established and verified. The three-dimensional finite element method was used to establish the models of anterior cervical compression fusion and internal fixation surgical implants and anterior cervical compression fusion and internal fixation combined with the partial resection of different amounts of the unilateral or bilateral uncovertebral joints. The models were tested under six working conditions: flexion, extension, left bending, right bending, left rotation, and right rotation. The surgical models were compared regarding the stress distribution of the titanium mesh, titanium plate and screw, and endplate, and the peak displacement of the vertebral body.

**Results:**

There were no significant differences in the stress distribution and peak displacement of the vertebral body of ACDF combined with different amounts of uncovertebral joint resection in the states of flexion and extension. However, there were significant increases in the stress distribution and peak displacement of the vertebral body in the states of left and right bending and rotation. In the states of left and right bending and rotation, the stress distribution and peak displacement of the vertebral body were significantly greater in the models with bilateral partial resection of the uncovertebral joints than in the models with unilateral partial resection of the uncovertebral joints. Bilateral resection of the uncovertebral joints by 30–40% and unilateral resection of the uncovertebral joints by 40–50% resulted in the greatest increases in the maximum stress distribution of the titanium plate and screw and the peak displacement of the vertebral body.

**Conclusion:**

Finite element analysis of the biomechanical changes in the cervical spine showed that anterior cervical compression fusion and internal fixation combined with bilateral resection of less than 30% of the uncovertebral joints or unilateral resection of less than 40% of the uncovertebral joints had little effect on the stability of the cervical spine.

## Background

Cervical spine radiculopathy (CSR) is the most common type of cervical spondylosis, with a reported prevalence of 1.07 to 1.76 per 1000 in males and 0.63 to 5.8 per 1000 in females [1]. CSR accounts for about 50–60% of cervical spondylosis cases and is more common in middle-aged and older adults. Chronic degeneration of the cervical spine is the basic cause of the occurrence and development of CSR. The main symptoms of CSR are neck pain, arm numbness, and limited neck movement, and some patients with CSR develop depression and insomnia, which seriously decrease quality of life [2]. For CSR that does not respond to conservative treatment, anterior cervical decompression and fusion (ACDF) is a routine and effective method that is considered the “gold standard” [3, 4]. ACDF effectively relieves nerve root compression, reconstructs the stability of the diseased segment, and restores the physiological curvature of the spine [5–7]. However, there is still some controversy regarding the optimal treatment of CSR with uncovertebral joint (UJ) hyperplasia, osteophyte formation, and root canal stenosis. ACDF reportedly achieves an ideal curative effect through indirect decompression of intervertebral distraction and fixation and fusion of the surgical segments. However, while ACDF combined with UJ resection quickly relieves nerve root compression and pain, the potential complications include large intraoperative blood loss, vertebral artery damage, and subsidence of the titanium mesh cage [8–12].

Although some studies have reported the use of ACDF combined with UJ resection for the treatment of CSR, there are few postoperative biomechanical studies [8, 9, 13–15]. No biomechanical studies have evaluated the relationship between the stability and mechanics of ACDF after the excision of different amounts of the uncinate processes, and the effect of partial excision of the uncinate processes on the stability of the spinal segment is still unclear. Therefore, we previously performed biomechanical experiments on seven fresh cadaveric adult cervical spine specimens (C3–C7) to test the three-dimensional (3D) range of motion after ACDF combined with different amounts of uncinate process excision. First, we performed ACDF combined with partial resection of the unilateral uncinate processes. Measurements showed that when the volume of the uncinate processes was reduced by 25.9% and the corresponding area of the uncinate articular surfaces was reduced by 35.6%, the cervical spine was reliably stable in all directions. Second, we performed ACDF combined with bilateral partial resection of the uncinate processes. When the volume of the uncinate processes was reduced by 45.9% and the corresponding area of the uncinate articular surfaces was reduced by 79.2%, the stability of the cervical spine worsened, especially during flexion. Third, we performed ACDF combined with bilateral total resection of the uncinate processes and found that the cervical spine was less stable in all directions. Overall, our previous study indicated that the stability of the cervical spine was consistent with those of clinical follow-up studies of ACDF combined with partial excision of the uncinate processes [8, 9, 11, 16]. However, our previous study had limitations, such as a small number of specimens, deviations in the processing of specimens, no evaluation of a 35.6–79.2% reduction in the articular surface area of the uncinate processes, and no clarification of the location of the stress concentrations of the upper and lower endplates and internal fixation structures.

With the continuous progress of numerical and computer technology, the finite element method has become an effective tool for investigating the biomechanics of the human spine [17]. Because finite element models have the correct anatomical structure, material properties, and boundary and load conditions to obtain reliable experimental results, many scholars have used this method to evaluate the biomechanical properties of the cervical spine [18–20]. Therefore, to overcome the abovementioned shortcomings of previous experimental studies, we established a simulation model of ACDF combined with different amounts of partial resection of the UJs and used the biomechanical 3D finite element method to analyze the von Mises stress distribution of the intervertebral cage, titanium plate and screw, and endplates, and the peak vertebral body displacement and subsidence of the interbody cage to judge the reliability and stability of cervical internal fixation. The present study will provide useful information for spine surgeons regarding the selection of the optimal operative procedures for CSR.

## Methods

### Establishment of the 3D model

1. Processing of CT data of the cervical spine
  After the provision of written informed consent, a healthy 34-year-old man (height 174 cm, weight 75 kg) underwent computed tomography (CT) scanning. Cervical deformity, tumor, infection, fracture, rheumatic disease, and cervical trauma were excluded by radiography, CT, and magnetic resonance imaging (MRI). Three-dimensional CT images of the cervical spine were extracted and used to establish a 3D finite element model of the normal healthy adult cervical spine. The participant was in supine position with the orthogonal positioning line on the midline of the body and the scanning table adjusted so that the scanning area was located in the scanning center. Sixty-four spiral slices were obtained from the upper edge of the C3 vertebral body to the lower edge of the C7 vertebral body. The data were saved in DICOM format and inputted into 3D reconstruction software (Mimics Medical 20.0, Materialise, Belgium). The following six directions were defined: anterior, posterior, left, right, top, and bottom. The threshold adjustment tool was used to extract the cervical spine bony contours in accordance with the CT gray values, the image definition was optimally adjusted, the appropriate threshold was determined, and a mask was generated. The hot area of the cervical spine was selected using the region growth tool, and the “modeling” command was executed to generate the cervical spine model. Selective editing and other tools were used to remove redundant burrs and fill the holes in the bones; this process was repeated and verified many times to establish the 3D model of the cervical spine, which was masked and saved in STL format (Fig. 1).

**Fig. 1:**
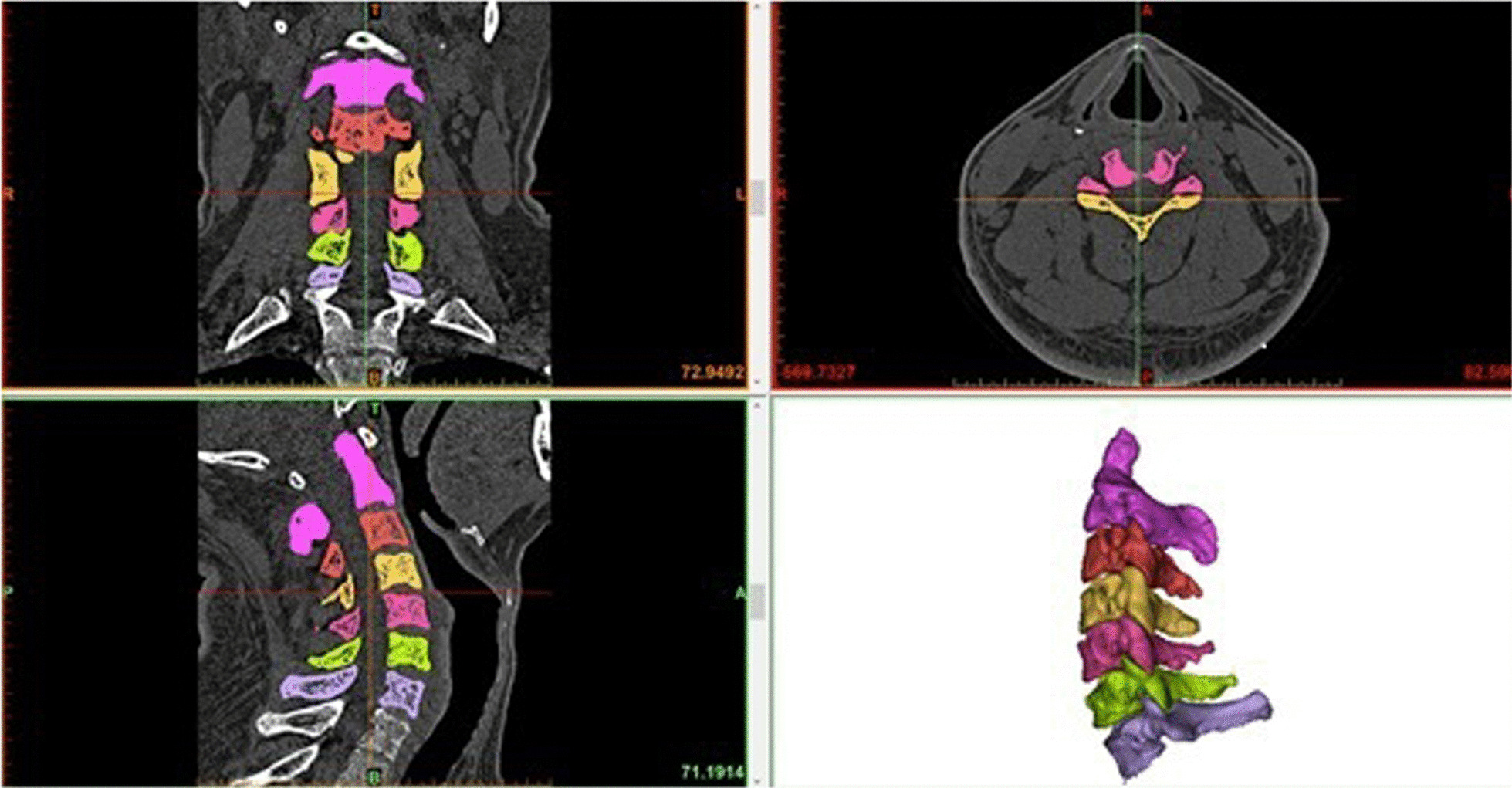
CT data file processing
2. Reverse reconstruction of the 3D solid model
  The STL file was imported into Geomagic software (Geomagic 2017, USA), and inverse reconstruction processing comprising denoising, polishing, and smoothing was performed to form a 3D image that was saved in IGES format (Fig. 2). Three-dimensional computer-aided design modeling software (Unigraphics NX12.0, Siemens PLM software) was used to establish structural models of the cortical bone, cancellous bone, endplates, nucleus pulposus, fibrous rings, and facet joints of the vertebral bodies, and a 3D solid model of C3–C7 was formed and saved in IGES format (Fig. 3).

**Fig. 2:**
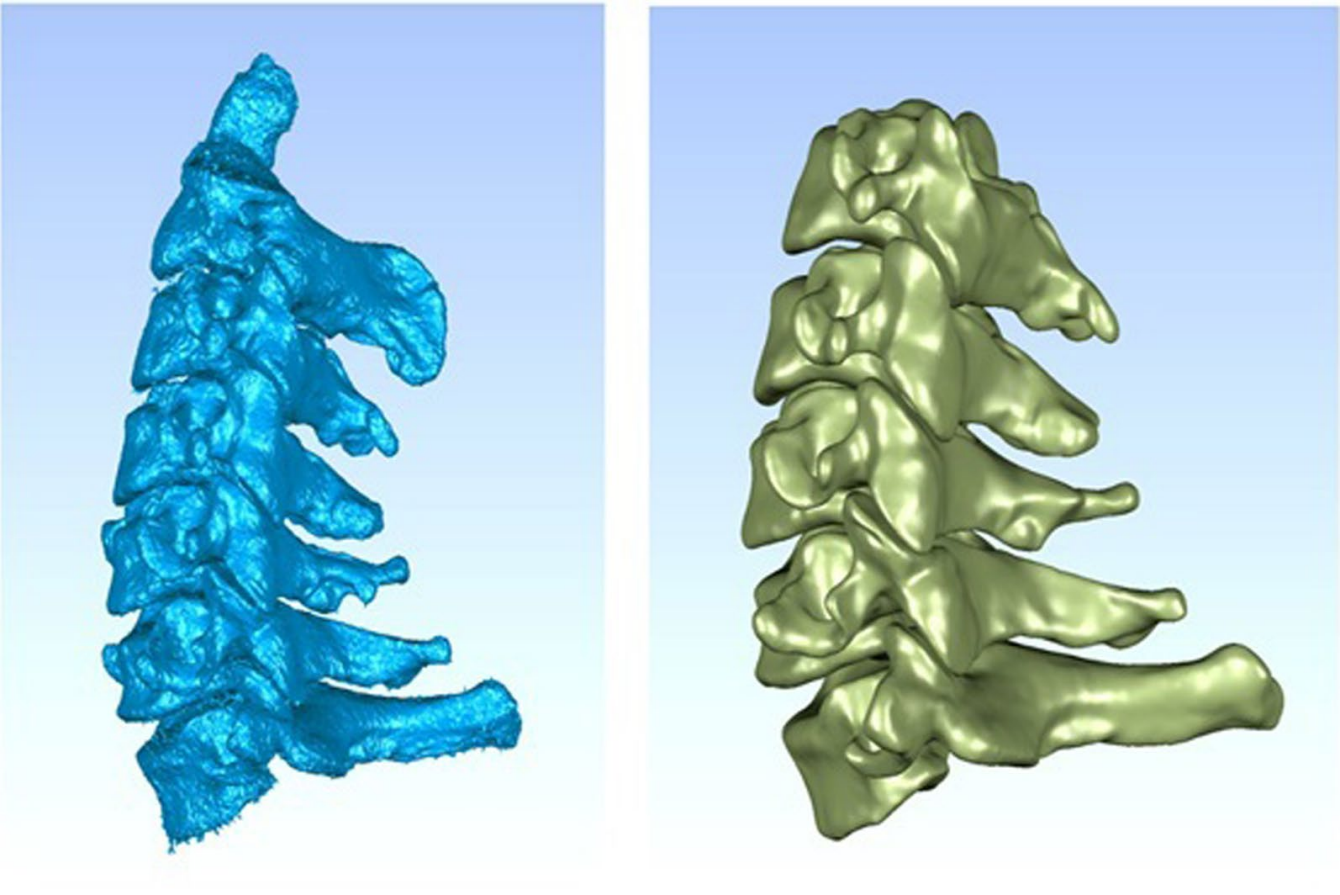
CT data file processing

**Fig. 3:**
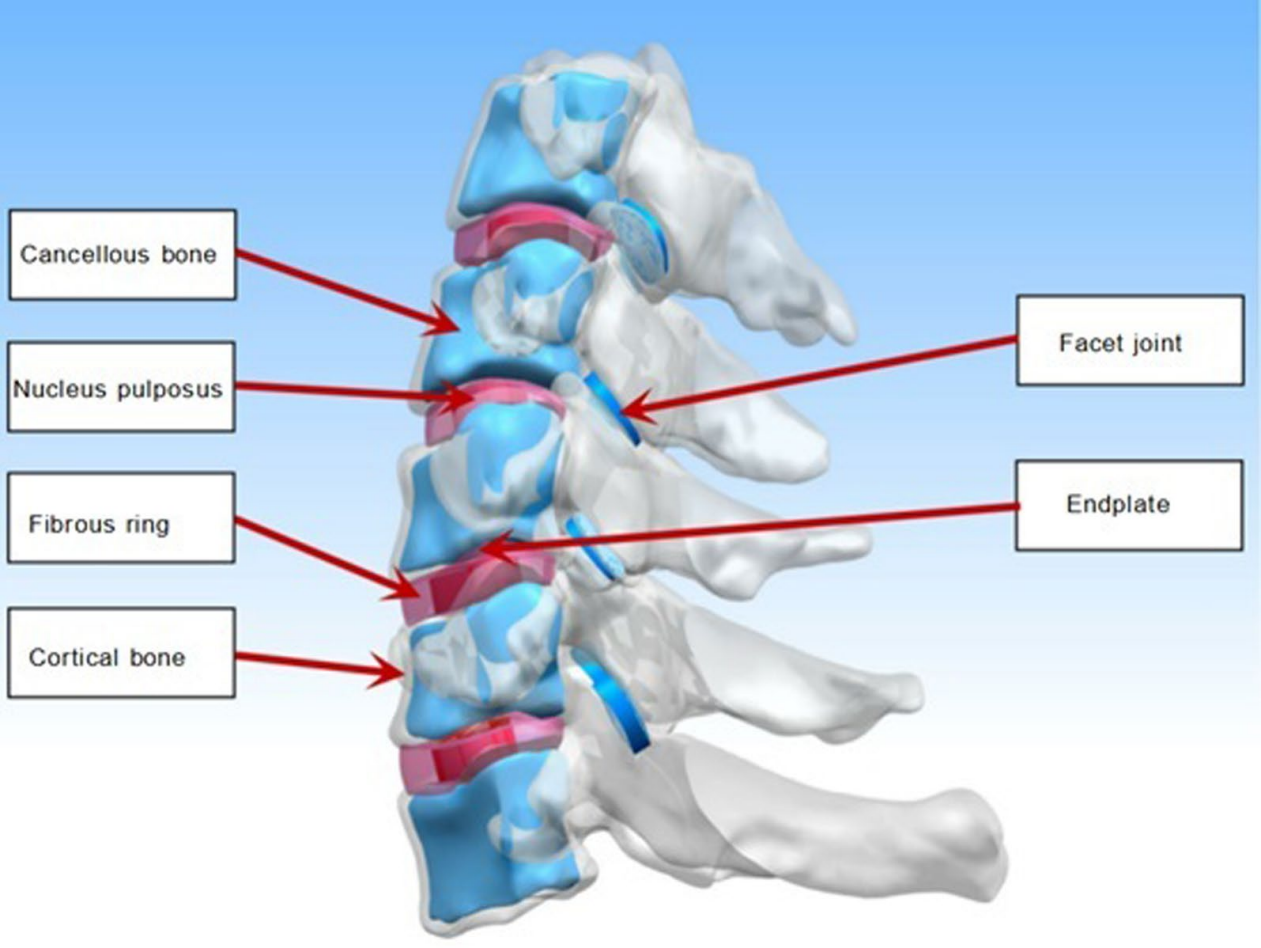
C3–C7 3D solid model of vertebral body
3. Meshing, assignment, and modeling completion.
  ANSYS Workbench 18.0 software (ANSYS, USA) was used to assign the assembled solid model, assign material parameters, set the contact, and assign each structure in accordance with the reference data (Table 1) [21]. Appropriate cell settings and mesh were then created (cell size 0.5–1.5 mm). The vertebral bodies, endplates, annulus fibrosus, nucleus pulposus, and facet joints were divided into 187 solid units, creating a 3D tetrahedron. The six intervertebral ligaments were the anterior longitudinal ligament, posterior longitudinal ligament, ligamentum flavum, interspinous ligament, supraspinous ligament, and capsular ligament. The adopted ligament unit was the Link180 unit, which was a 3D rod unit set to be only under tension, not compression, and the contact between the end point and the vertebral body was set as binding. The contact of joint capsule connecting part was set as frictionless surface contact, and the contact between intervertebral disk and vertebral endplate, intervertebral disk and vertebral cortical bone was set as binding. Finite element meshing, assignment, and other processing procedures were performed to complete the modeling.

**Table 1:** Material properties of lumbar finite element model

Material science	Elastic modulus/MPa	Poisson ratio	Cross-sectional area (mm^2^)
Cortical bone	12,000	0.29	–
Cancellous bone	450	0.29	–
Fibrous ring	3.4	0.4	–
Nucleus pulposus	1	0.49	–
Endplate	500	0.4	–
Facet joint	32.9	0.4	–
ALL	10	0.3	6.0
PLL	10	0.3	5.0
LF	1.5	0.3	5.0
ISL	1.5	0.3	10.0
SSL	1.5	0.3	5.0
CL	10	0.3	46
Titanium alloy	116,000	0.3	–
4. Grid sensitivity analysis (convergence analysis).
  After meshing the vertebral bodies, endplates, annulus fibrosus, nucleus pulposus and facet joints, the convergence analysis was carried out. For the analysis of results with different grid sizes of 0.5 mm, 1.5 mm, and 2.5 mm, the relative errors of most results are less than 5%, and the relative errors of all results are less than 10%, which is acceptable. According to this model, the mesh unit size is selected as 1.5 mm, and the model has 388,648 nodes and 247,307 units (Fig. 4).

**Fig. 4:**
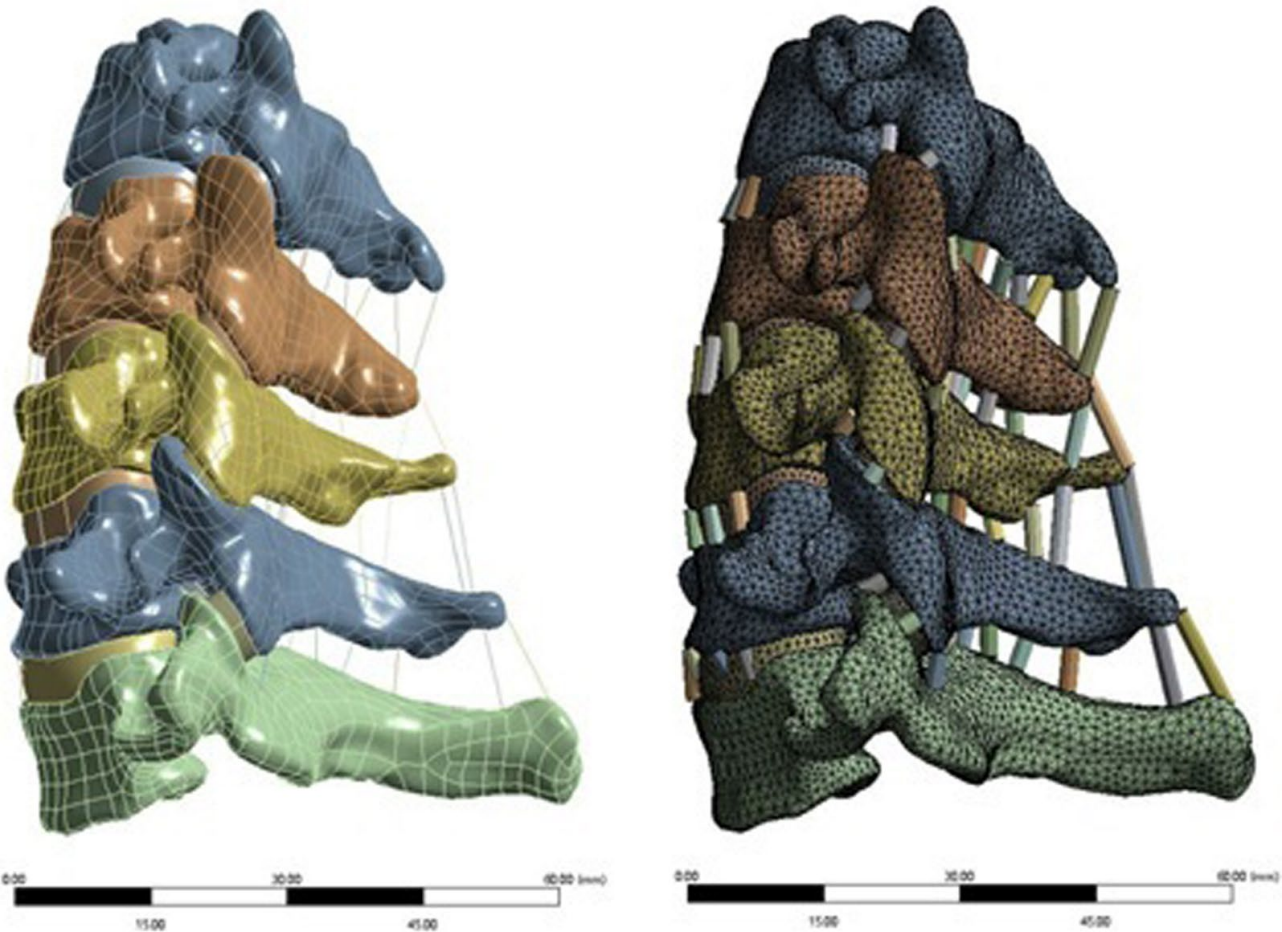
C3–C7 mesh generation model of vertebral body

### Verification of the 3D finite element model

1. Three-dimensional finite element model of C3–C7.
  The model was moved in different directions while simulating its own weight and additional torque. After calculation, the activities and ranges of the C3/4, C4/5, and C6/7 segments of this 3D finite element model under six working conditions of flexion, backward extension, left and right lateral flexion, and left and right rotation were compared with the findings of previous studies to test the effectiveness of the model. The boundary conditions and load conditions of the model were established in accordance with the biomechanical characteristics, physical motion characteristics, load conditions, and previous research data of the cervical spine. The boundary condition was that the motion degree of freedom of the lower surface of C7 was zero, and no boundary conditions were set for C3–C6 to enable this portion of the spine to bear the load. The load condition was that the preload of the model was 40 N on the upper surface of C3, and the additional motion torque was 8 Nm, so that the model could perform activities such as flexion and extension, lateral flexion, and rotation (Fig. 5).

**Fig. 5:**
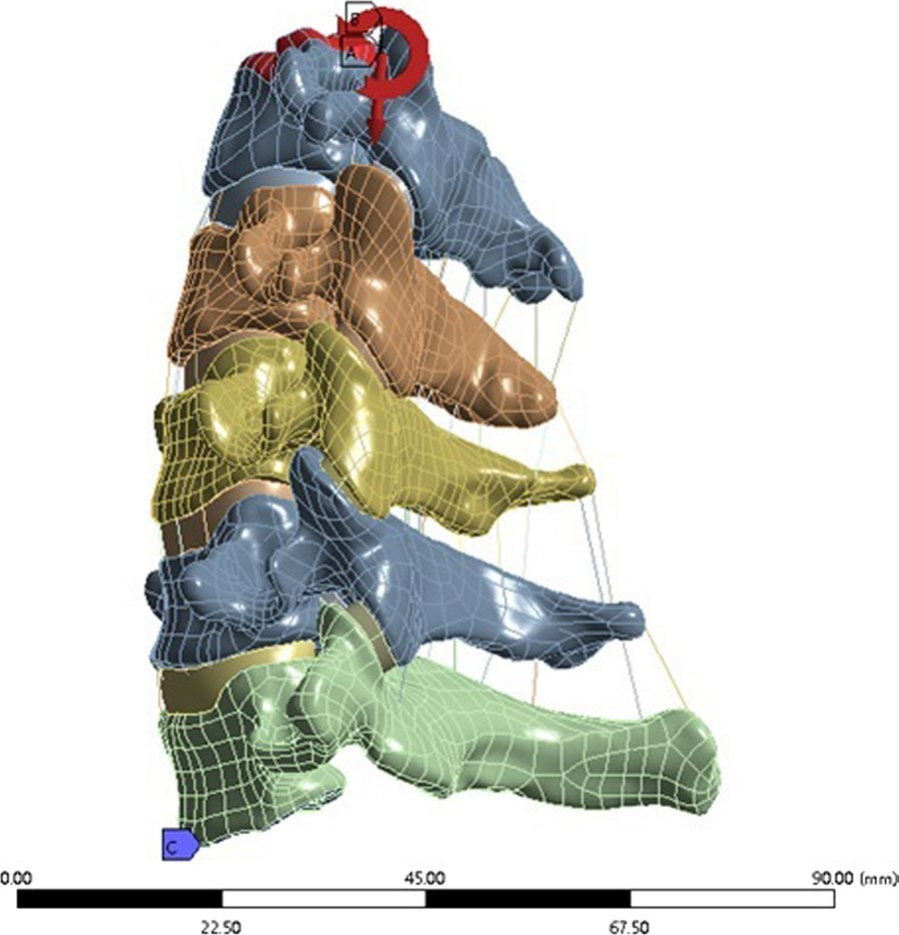
C3–C7 vertebral body model load and constraint setting
2. C3–C7 3D finite element model test.
  The CT data, modeling software, and finite element analysis software were used to establish the 3D finite element model of C3–C7 in normal healthy adults. The model reflected the mechanical characteristics of the cervical spine, with different material characteristics of different tissues and two types of units (the Solid187 solid unit and Link180 unit) to simulate the cortical bone, cancellous bone, posterior bony structures, fibrous rings, nucleus pulposus, endplates, and ligaments of the vertebral bodies, with a total of 388,648 nodes and 247,307 units.
  Model validation was performed under a 40 N preload and 1.5 Nm additional motion torque with six degrees of freedom in flexion, extension, lateral flexion, and rotation, and the range of motion of each segment was compared with previous studies (Fig. 6). The results of the model validation are shown in Table 2. The range of motion of the finite element model was within the scope of the reference models, verifying that the C3–C7 vertebral body finite element model established in the present study was appropriate for further finite element analysis.

**Fig. 6:**
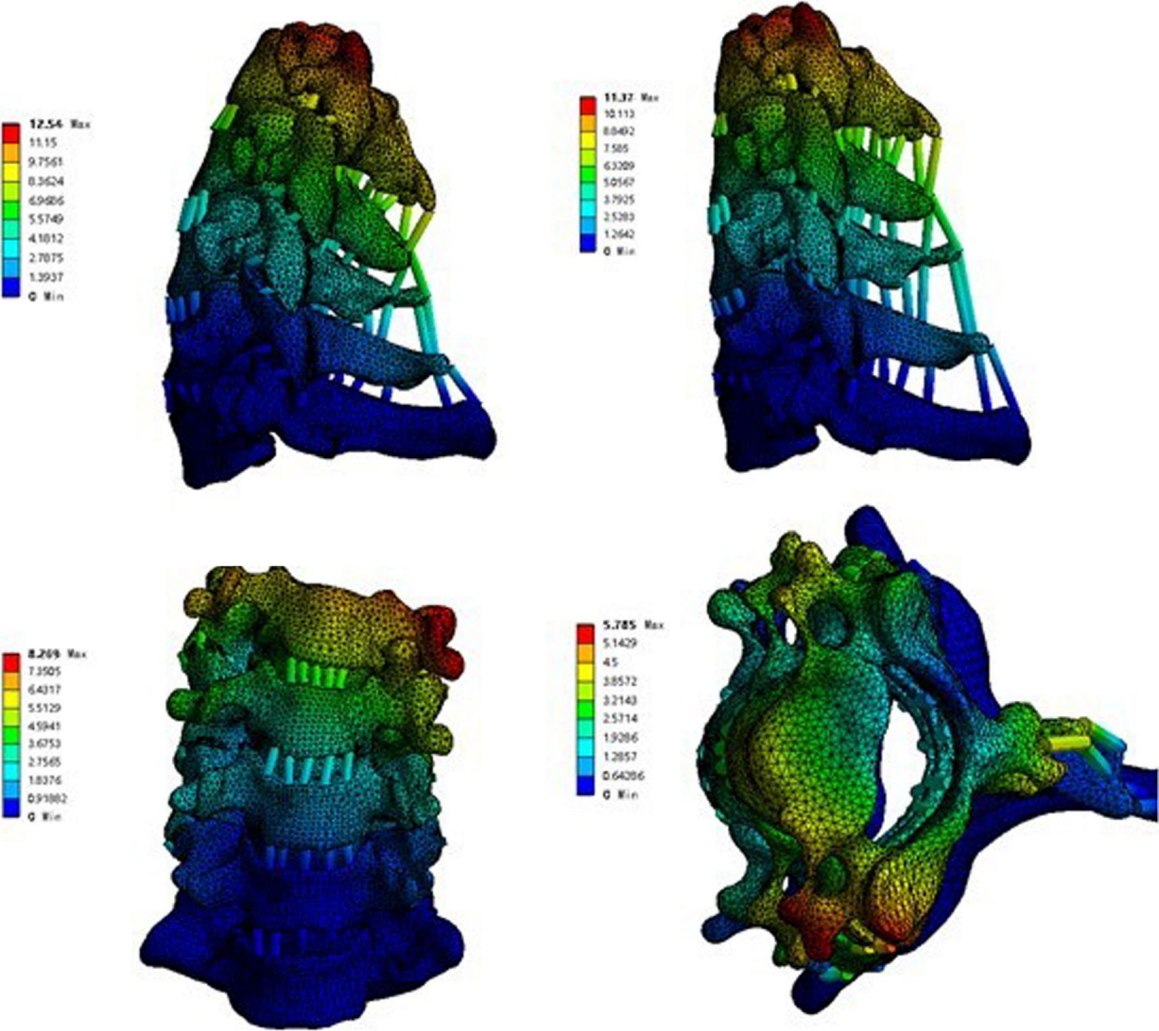
Test results of C3–C7 vertebral body in flexion, extension, bending, and rotation

**Table 2:** Comparison results between C3–C7 three-dimensional finite element model and literature data (unit: degree)

Working condition	Working condition	This study	Punjabi et al. (Literature)
Anteroposterior flexion	C3–4	8.5	7.3–11.5
	C4–5	8.8	7.4–10.1
	C5–6	8.3	7.2–9.9
	C6–7	8.9	5.7–11.5
Left and right lateral flexion	C3–4	6.7	3.4–15.4
	C4–5	6.3	3.4–15.4
	C5–6	6.6	3.1–15.4
	C6–7	7.2	3.4–15.4
Left and right rotation	C3–4	5.1	2.3–13.0
	C4–5	5.6	2.3–13.6
	C5–6	6.1	2.3–13.8
	C6–7	5.7	2.1–10.8

### Three-dimensional finite element analysis of ACDF combined with different amounts of partial resection of the UJs

**Fig. 7:**
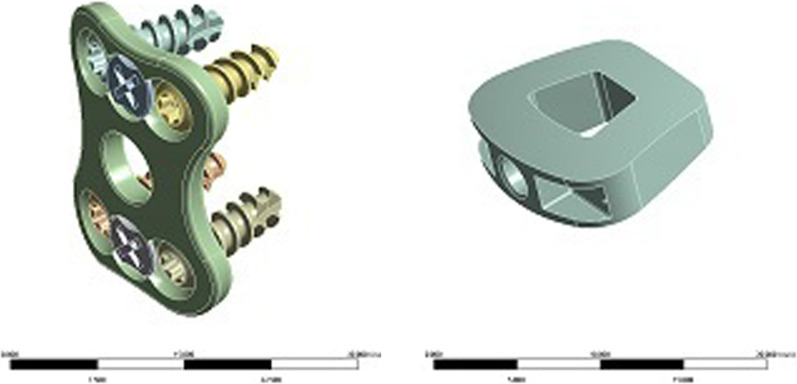
ACDF surgical implant (intervertebral fusion cage, titanium plate, screw) model

**Fig. 8:**
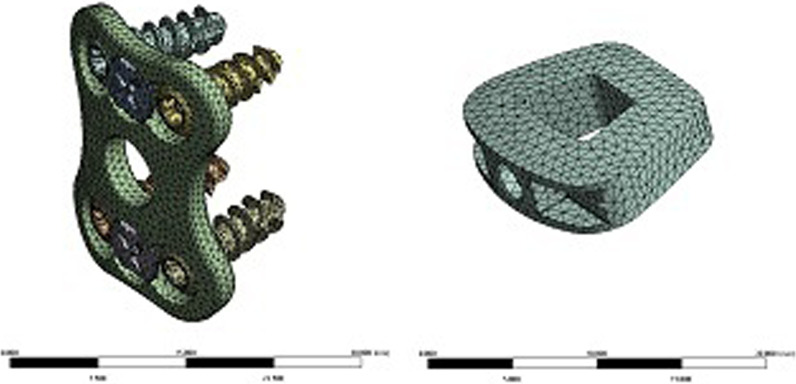
Grid model of ACDF intraoperative plants (intervertebral fusion cage, titanium plate, screw)

1. Establishment of finite element models of ACDF surgical implants (intervertebral fusion cage, titanium plate, screw)
  The design drawings of the intervertebral fusion cage, titanium plate, and screw were entered into the 3D drawing software NX12.0, and 3D geometric models were created using the functions of stretching, cutting, and rounding (Fig. 7). The model was imported into ANSYS Workbench 18.2 software to obtain the meshing model of the ACDF implants (intervertebral fusion cage, titanium plate, screw) (Fig. 8). Two fusion material properties were set (Table 1).
2. Establishment of a finite element model of the cervical spine with ACDF combined with different amounts of partial resection of the UJs

**Fig. 9:**
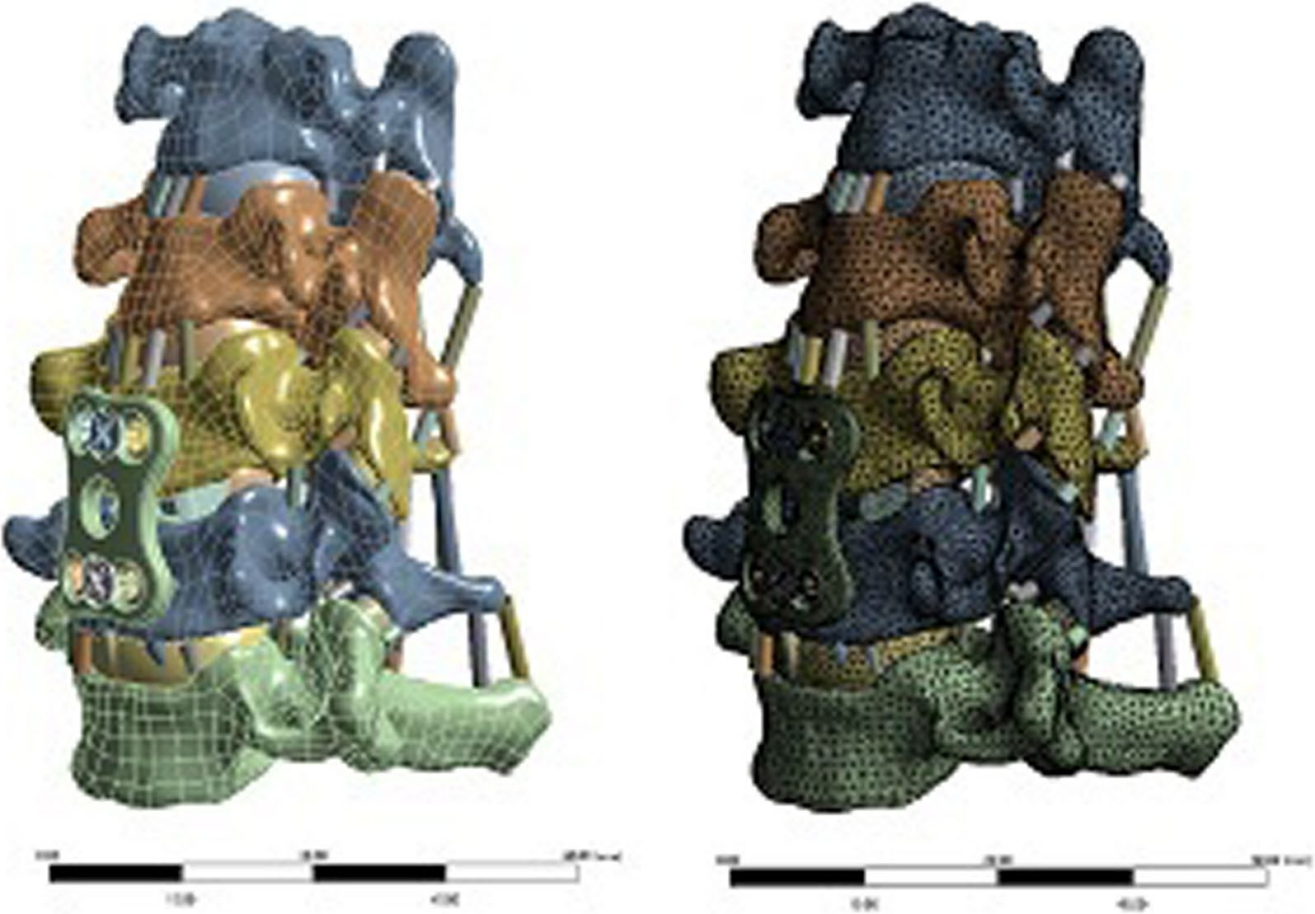
Finite element model of cervical spine with ACDF combined with different amounts of uncovertebral joint resection

The established C3–C7 vertebral body model was combined with the implant model to simulate the surgical process of ACDF. A cervical geometric model of ACDF combined with different amounts of partial resection of the uncinate vertebral joints was obtained. The overall model was then imported into ANSYS Workbench 18.2. Material attribute distribution and meshing were performed, and the establishment of the whole finite element model was completed (Fig. 9).

Similarly to the method used to verify the effectiveness of the model, the degree of freedom of the lower surface of the C7 vertebral body was constrained, and 40 N of downward preload was applied on the upper surface of C3 to simulate the upper body weight; 1.5 Nm of moment loading was added to simulate flexion, backward extension, and left and right lateral flexion, while 1.5 Nm of torque was added to simulate left and right rotation. The von Mises stress distribution and peak displacement characteristics of the vertebral body on the intervertebral fusion cage, endplates, titanium plate, titanium nail, and UJs after different amounts of UJ resection under six working conditions were analyzed, and the influence of different amounts of UJ resection on the stability of the cervical spine was evaluated.

## Results

The 3D modeling software was used to establish a finite element model of the cervical spine after ACDF combined with uncinate vertebral joint partial resection, with a total of 533,450 nodes and 339,750 elements. According to the principle of anterior cervical spine surgery, the C3–C7 model and ACDF surgical implants were combined to obtain the 3D finite element model of ACDF surgery.

### Interbody fusion cage contact pressure

**Fig. 10:**
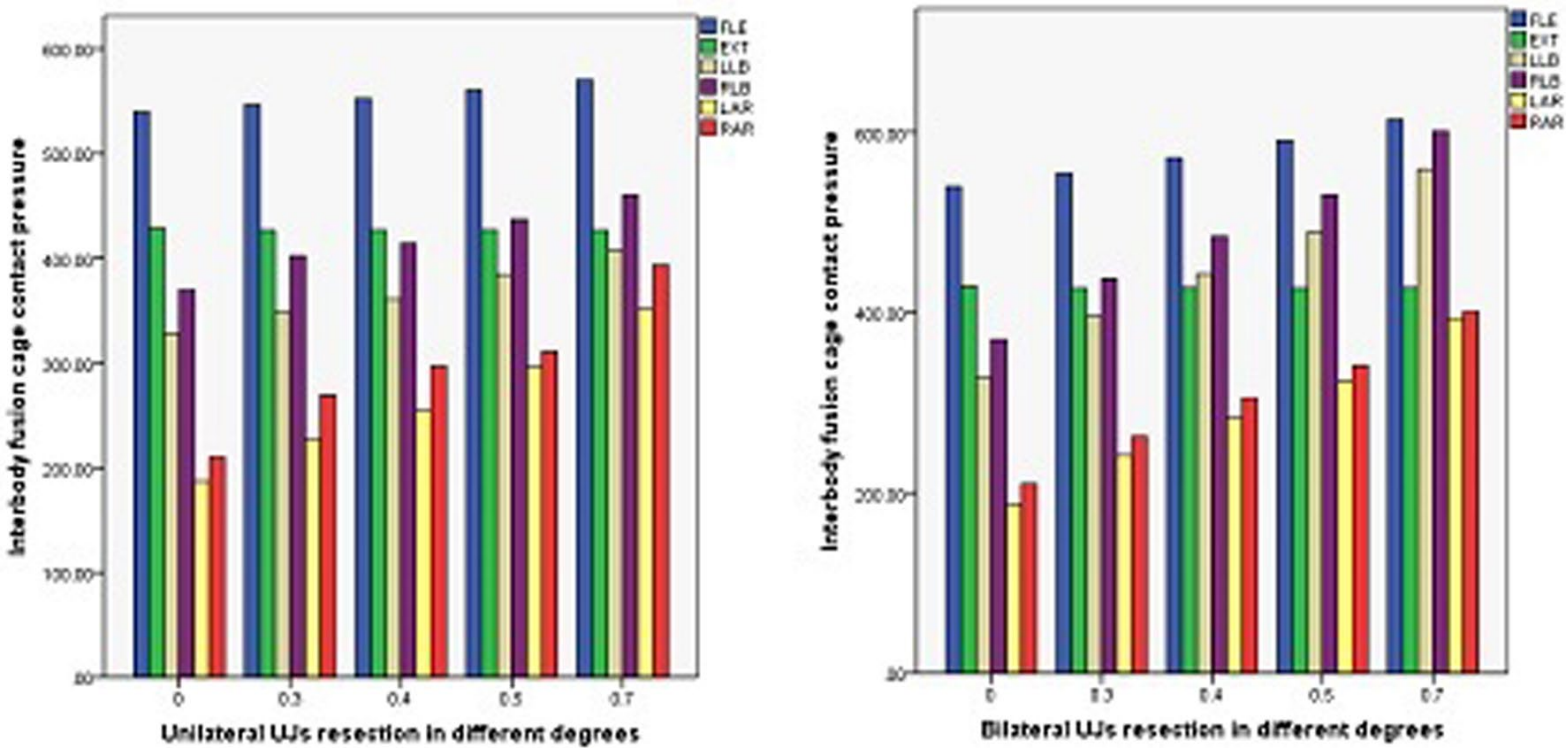
Contact pressure distribution of interbody cage in different amounts of unilateral and bilateral uncovertebral joint resection

**Fig. 11:**
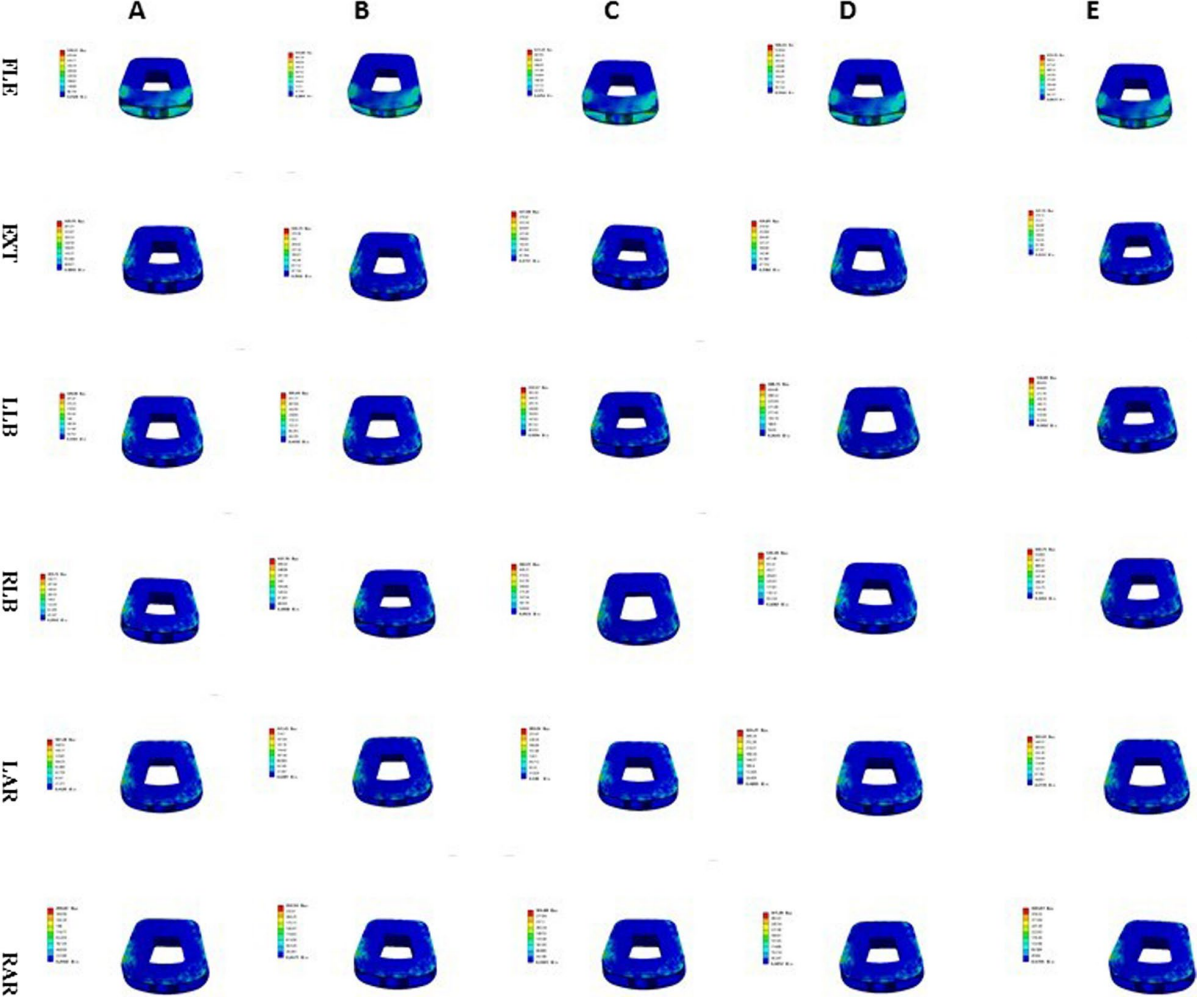
SStress nephogram of fusion cage of ACDF combined with bilateral uncovertebral joints in different amounts of partial resection model in different motion states. **A**–**E** represents 0%, 30%, 40%, 50% and 70% of uncinate vertebrae joints were removed respectively

**Fig. 12:**
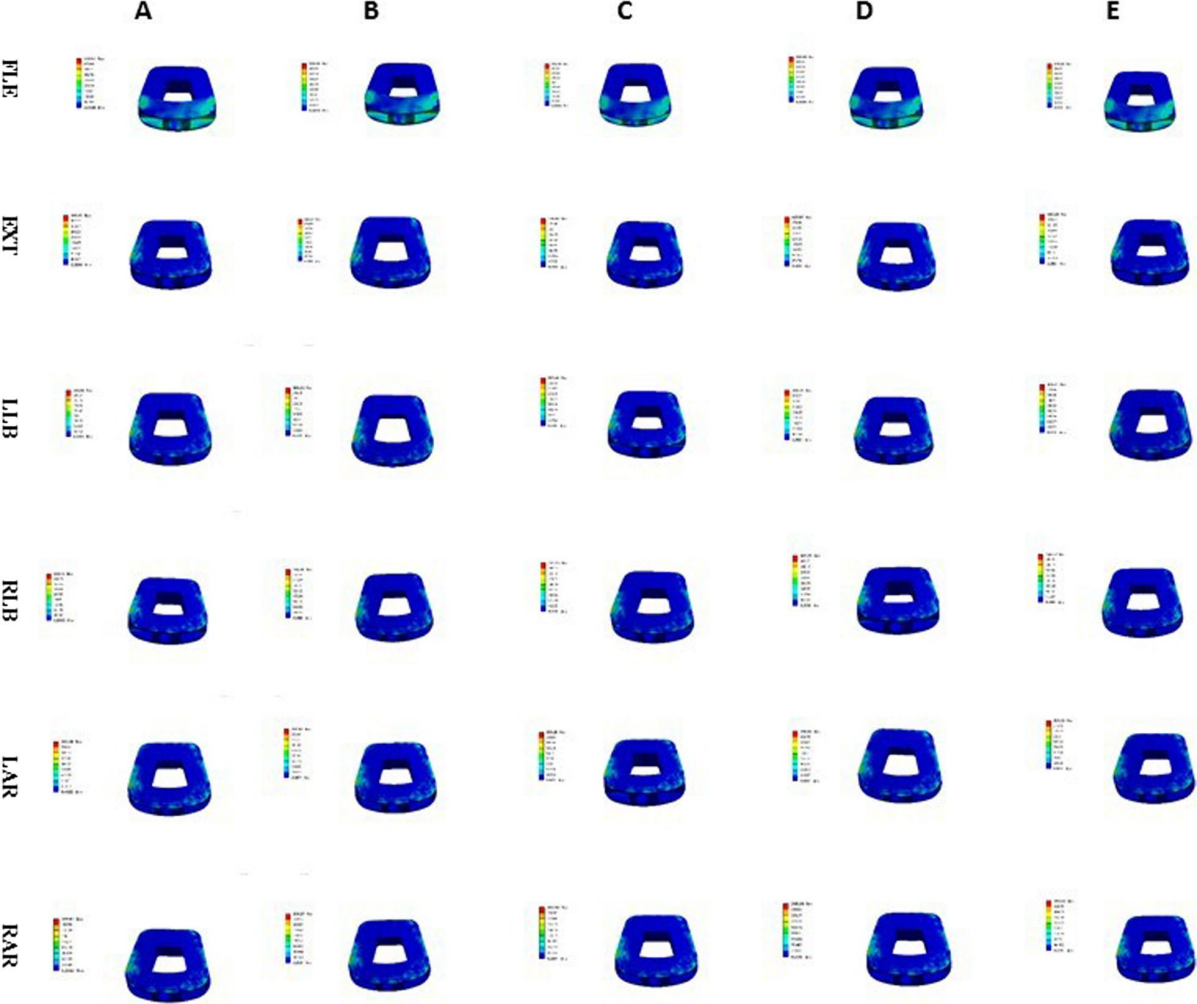
Stress nephogram of fusion cage of ACDF combined with unilateral uncovertebral joints in different amounts of partial resection model in different motion states. **A**–**E** represents 0%, 30%, 40%, 50% and 70% of uncinate vertebrae joints were removed respectively

The interbody fusion cage contact pressures of ACDF combined with different amounts of UJ partial resection are shown in Figs. 10, 11, and 12. In the model with bilateral UJ resection, as the amount of UJ resection was increased, the maximum stress of the interbody cage increased the most when the cervical vertebrae were laterally flexed and rotated; the maximum stress of the interbody cage increased by 12.09%, 41.30%, 38.45%, 52.37%, and 47.64% in flexion, left and right bending, and left and right axial rotation, respectively. However, in the models with different amounts of unilateral UJ resection, as the amount of UJ resection was increased, the maximum stress of the interbody cage increased greatly when the cervical vertebrae were laterally flexed and rotated, especially during left and right rotation; the maximum stress of the cage increased by 5.39%, 19.47%, 19.69%, 46.75%, and 46.66% in flexion, left and right bending, and left and right axial rotation, respectively. In both models, the maximum stress of the interbody cage did not change significantly in extension with increasing UJ resection.

### Endplate contact pressure

**Fig. 13:**
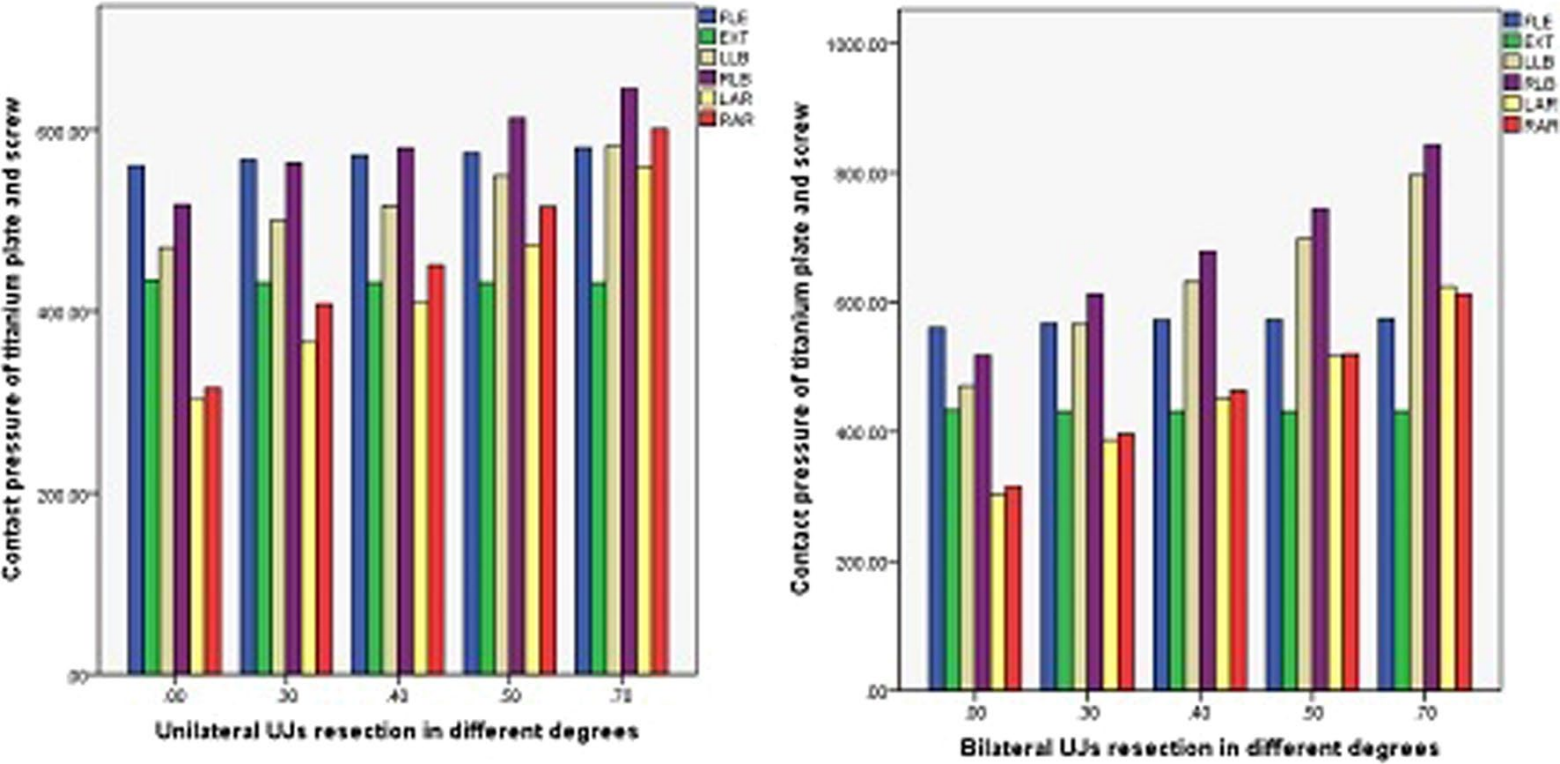
Contact pressure distribution of End plate in different amounts of unilateral and bilateral uncovertebral joint resection

**Fig. 14:**
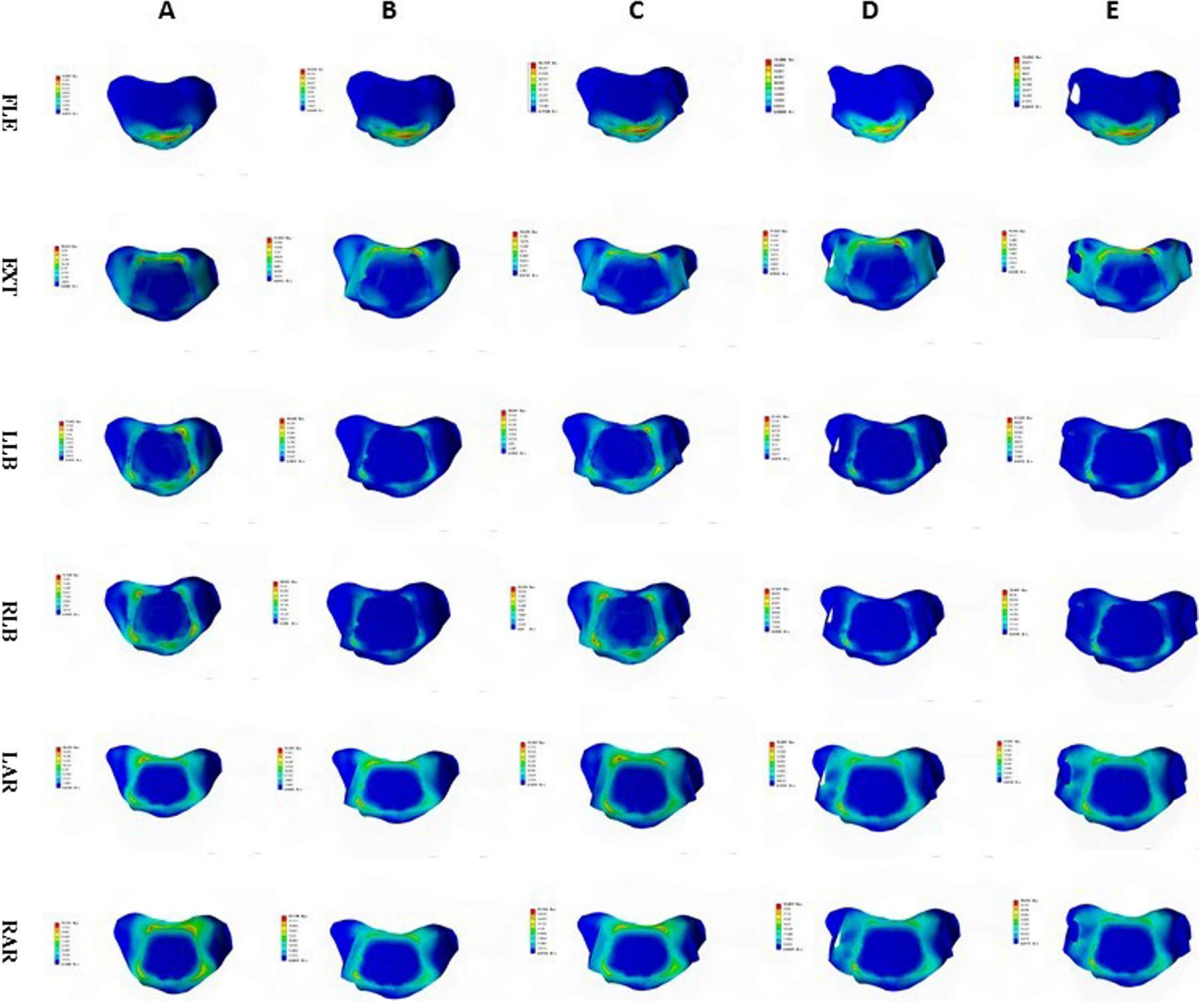
Stress nephogram of end plate of ACDF combined with bilateral uncovertebral joints in different amounts of partial resection model in different motion states. **A**–**E** represents 0%, 30%, 40%, 50% and 70% of uncinate vertebrae joints were removed respectively

**Fig. 15:**
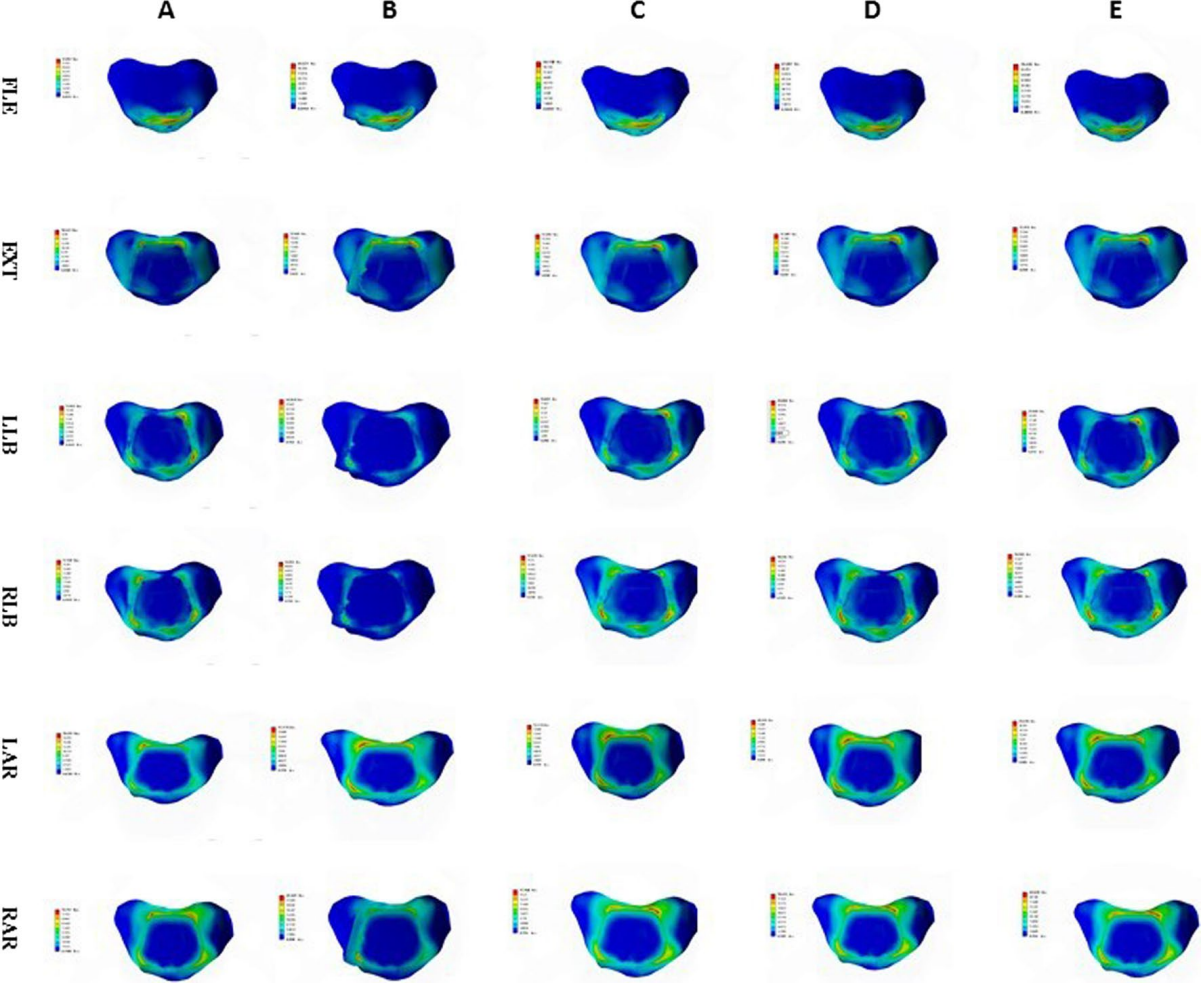
Stress nephogram of End plate of ACDF combined with unilateral uncovertebral joints in different amounts of partial resection model in different motion states. **A**–**E** represents 0%, 30%, 40%, 50% and 70% of uncinate vertebrae joints were removed respectively

The endplate contact pressures of ACDF combined with different amounts of UJ partial resection are shown in Figs. 13, 14, and 15. The comparison of the two models of ACDF combined with different amounts of unilateral and bilateral UJ resection showed that the maximum endplate contact pressure of the bilateral UJ resection model had different increases in each motion state than the simple model, except in the extension state. In ACDF combined with different amounts of bilateral UJ resection, the maximum stress of the endplate contact in flexion, left and right bending, and left and right rotation increased by 11.40%, 74.74%, 77.67%, 56.29%, and 67.83%, respectively; in ACDF combined with different amounts of unilateral UJ resection, the maximum stress of the endplate contact in flexion, left and right bending, and left and right rotation increased by 7.92%, 22.35%, 12.35%, 31.31%, and 43.66%, respectively.

### Contact pressure of the titanium plate and screw

**Fig. 16:**
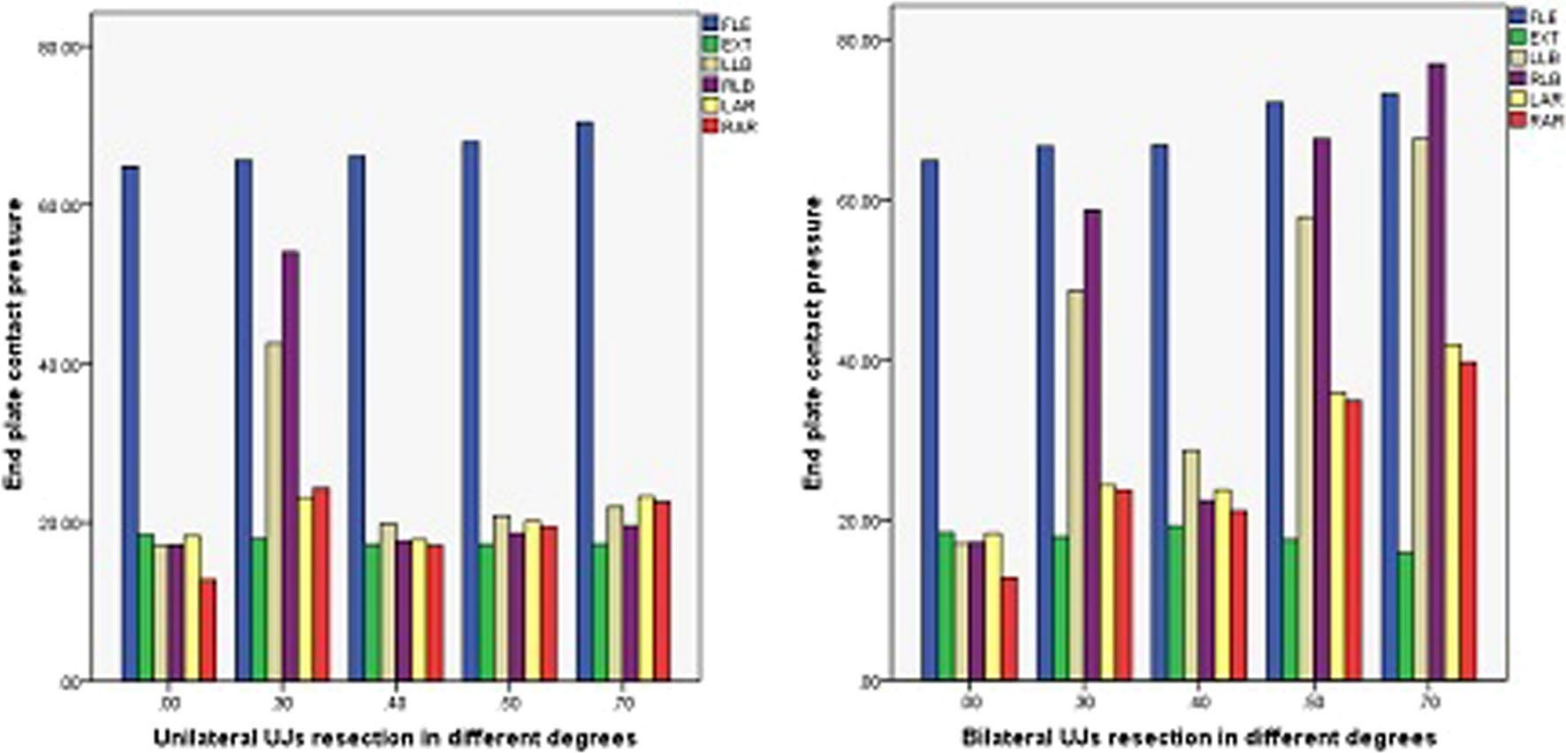
Contact pressure distribution of titanium plate and screw in different amounts of unilateral and bilateral uncovertebral joint resection

**Fig. 17:**
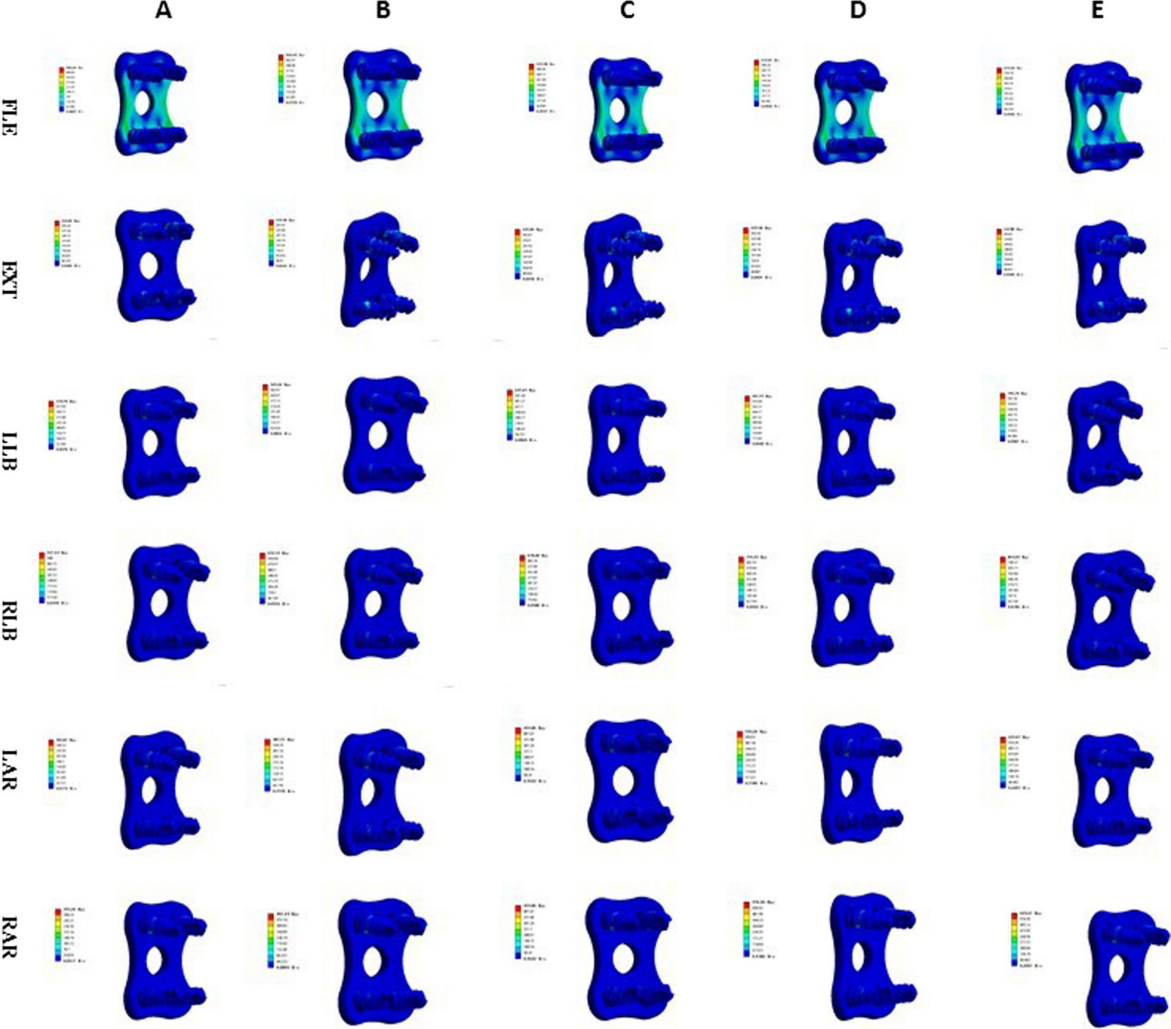
Stress nephogram of titanium plate and screw of ACDF combined with bilateral uncovertebral joints in different amounts of partial resection model in different motion states. **A**–**E** represents 0%, 30%, 40%, 50% and 70% of uncinate vertebrae joints were removed respectively

**Fig. 18:**
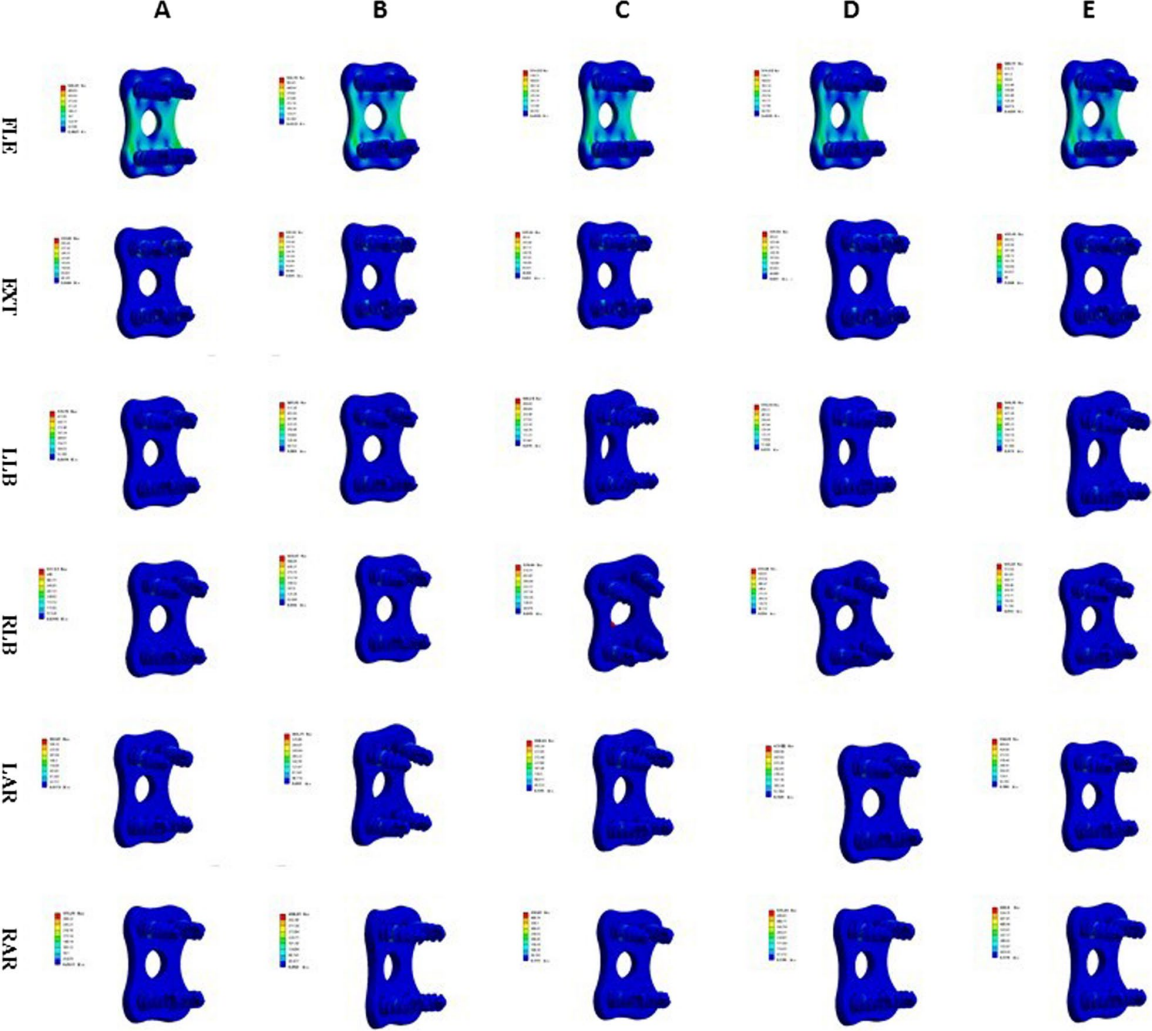
Stress nephogram of titanium plate and screw of ACDF combined with unilateral uncovertebral joints in different amounts of partial resection model in different motion states. **A**–**E** represents 0%, 30%, 40%, 50% and 70% of uncinate vertebrae joints were removed respectively

The contact pressure of the titanium plate and screw in ACDF combined with different amounts of partial resection of the UJs is shown in Figs. 16, 17, and 18. In both ACDF combined with bilateral UJ resection and ACDF combined with unilateral UJ resection, the contact pressures of the titanium plate and screw were greatly increased in left and right bending and rotation, especially in left and right rotation. In ACDF combined with different amounts of bilateral UJ resection, the contact pressure of the titanium plate and screw in left and right bending and left and right rotation increased by 38.12%, 38.61%, 51.44%, and 48.52%, respectively; in ACDF combined with different amounts of unilateral UJ resection, the contact pressure of the titanium plate and screw in left and right bending and left and right rotation increased by 19.20%, 19.79%, 45.79%, and 47.54%, respectively. The contact pressure of the titanium plates and screw in ACDF combined with unilateral and bilateral UJ resection increased slightly in flexion, but did not change significantly in extension.

### Maximum displacement of the cervical vertebrae

**Fig. 19:**
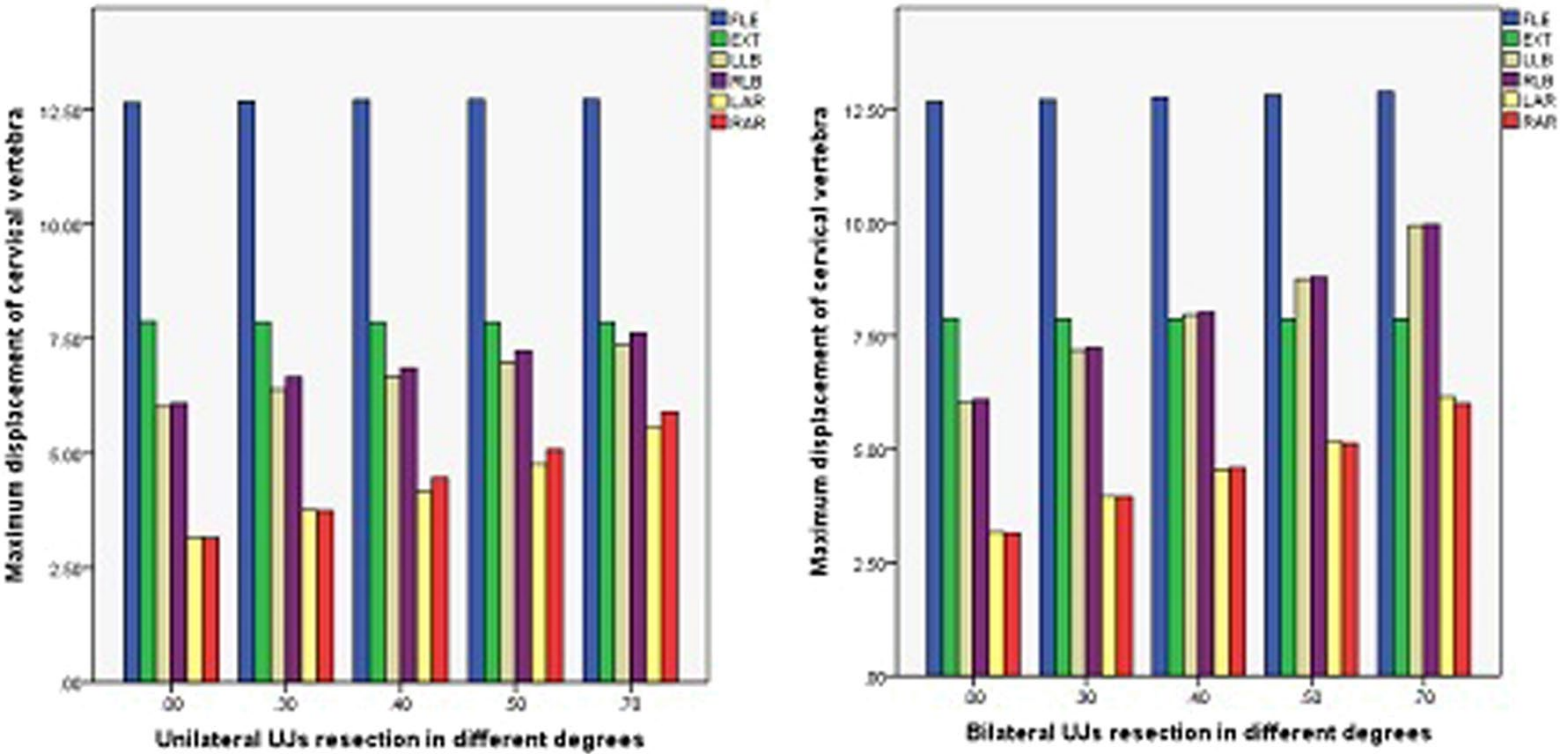
Maximum displacement of cervical vertebra in different amounts of unilateral and bilateral uncovertebral joint resection

**Fig. 20:**
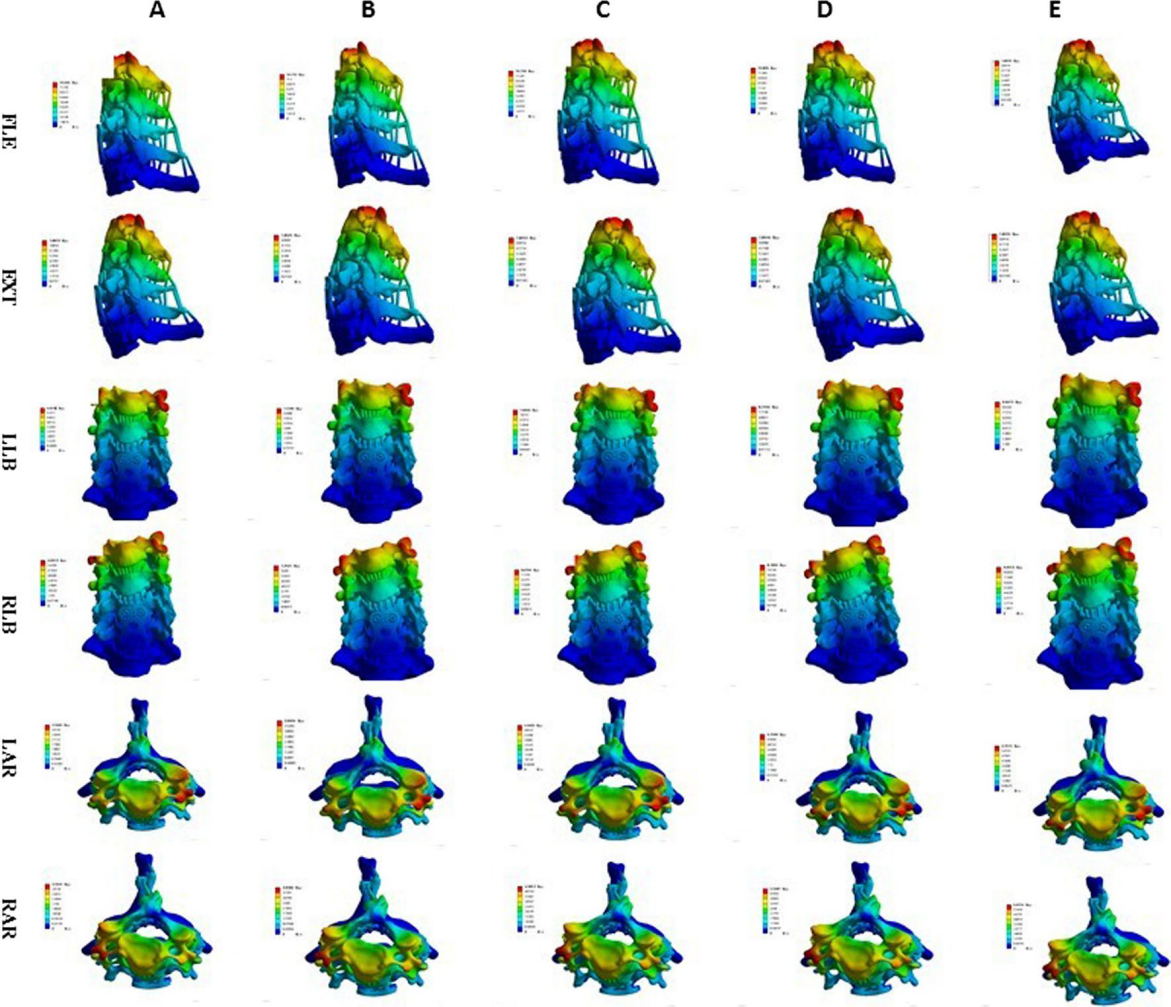
Stress nephogram of cervical vertebra displacement of ACDF combined with bilateral uncovertebral joints in different amounts of partial resection model in different motion states. **A**–**E** represent 0%, 30%, 40%, 50%, and 70% of uncinate vertebrae joints were removed, respectively

**Fig. 21:**
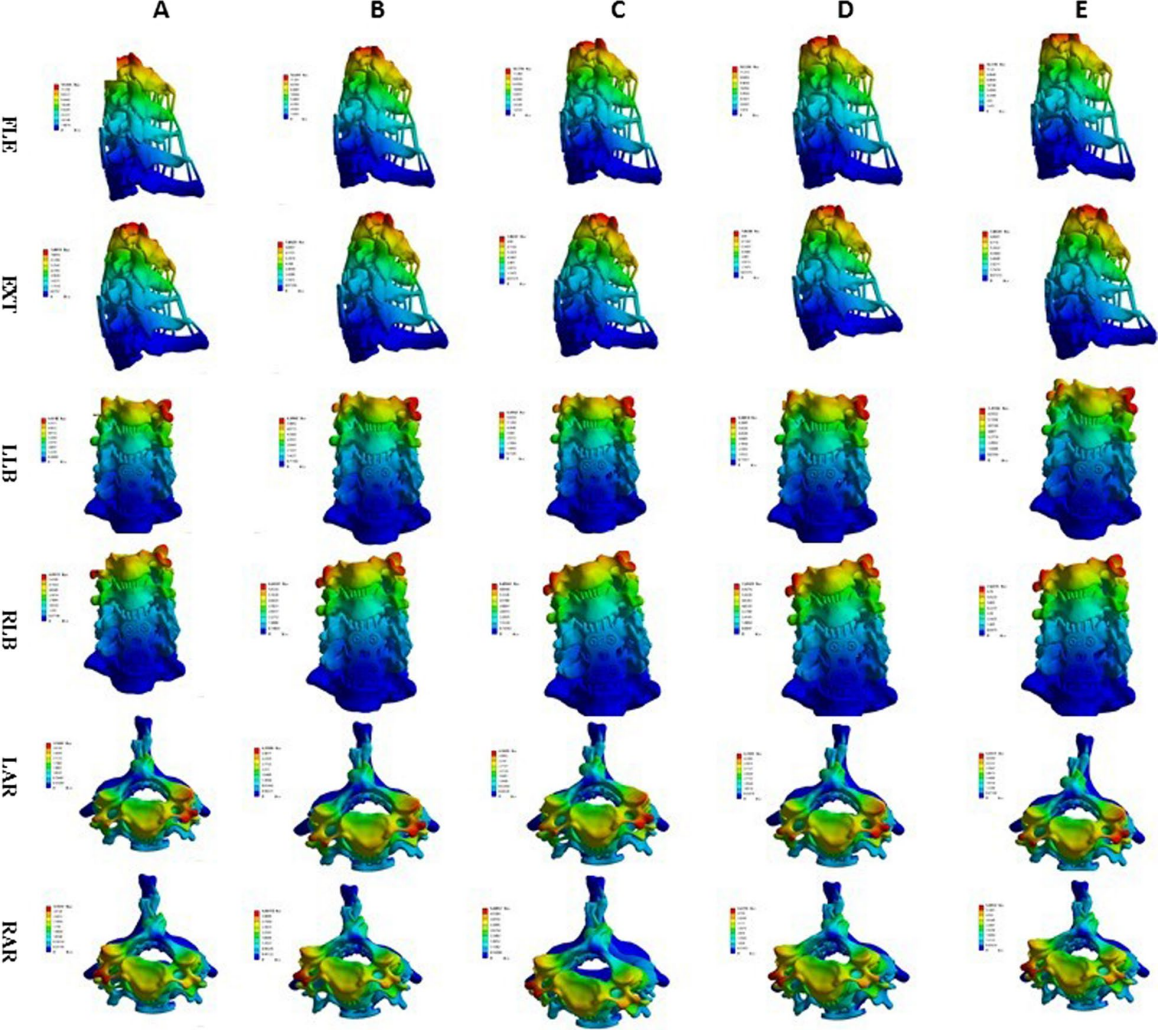
Stress nephogram of cervical vertebra displacement of ACDF combined with unilateral uncovertebral joints in different amounts of partial resection model in different motion states. **A**–**E** represent 0%, 30%, 40%, 50%, and 70% of uncinate vertebrae joints were removed, respectively

The maximum displacement of the cervical vertebrae in the models of ACDF combined with different amounts of partial resection of the UJs is shown in Figs. 19, 20, and 21. In ACDF combined with different amounts of bilateral UJ resection, the maximum displacement of the cervical vertebrae in left and right bending and left and right rotation increased by 39.37%, 38.69%, 48.54%, and 47.42%, respectively; in ACDF combined with different amounts of unilateral UJ resection, the maximum displacement of the cervical vertebrae increased by 18.30%, 20.05%, 42.99%, 31.31%, and 46.53%, respectively. The increase in the maximum displacement of the cervical vertebrae was smaller in ACDF with unilateral UJ resection than in ACDF with bilateral UJ resection.

## Discussion

The present study used an indirectly validated 3D finite element model of a normal C3–C7 segment to simulate several anterior cervical surgeries, including ACDF alone, ACDF with unilateral and bilateral 30% UJ resection, ACDF with unilateral and bilateral 40% UJ resection, ACDF with unilateral and bilateral 50% UJ resection, and ACDF with unilateral and bilateral 70% UJ resection. The comparison and analysis of the biomechanical changes in the nine surgical models will aid in the selection of an appropriate surgical plan in clinical practice. Previous biomechanical studies have assessed the interbody fusion cage contact pressure, endplate contact pressure, titanium plate and screw contact pressure, and displacement of the cervical vertebrae [13, 22–24]. However, the biomechanical characteristics of the cervical spine after various anterior cervical surgeries have not been clarified. Therefore, we constructed a finite element model of ACDF combined with different amounts of UJ resection and calculated the peak contact pressure of the interbody fusion cage, endplate, and titanium plate and screw, and the postoperative displacement of the cervical vertebrae.

The interbody fusion cage contact pressure was greatest in the model of ACDF with bilateral 70% UJ resection. The maximum interbody fusion cage contact pressure was detected in the flexion state; however, as the amount of UJ resection was increased, the pressure of the interbody fusion cage increased the most in the lateral flexion state, followed by the rotation states. We speculated that this might be because the UJs, which are load-bearing structures, have a close relationship with the intervertebral disks [25]. We also observed that the interbody fusion cage contact pressure was greater in the bilateral UJ resection models than the unilateral UJ resection models, indicating that the UJs play a role in stabilizing the cervical spine. The stability of the cervical spine worsened after excessive UJ resection, especially in the states of lateral flexion and rotation, indicating that the UJs also play an important role in limiting lateral flexion and rotation [26]. These findings suggest that patients should wear a neck brace as much as possible to limit the lateral flexion and rotation of the neck after anterior cervical spine surgery to prevent stress concentration, especially when the UJs are partially removed.

The endplate contact pressure was greatest in the flexion state. There was only a small change in the endplate contact pressure in the lateral flexion and rotation states after ACDF with 40%, 50%, and 60% unilateral resection of the UJs. However, in the models of ACDF with bilateral UJ resection, the endplate contact pressure changed greatly. In the model with bilateral 40% UJ resection, the endplate contact pressure increased the most in the lateral flexion state, followed by the rotation states. The significant change in endplate contact pressure after bilateral 40% UJ resection indicates the occurrence of stress concentration. These findings show that the stability of the cervical spine was significantly worse after ACDF with bilateral 40% UJ resection, while unilateral UJ resection had no significant effect on the stability of the cervical spine. This is consistent with the findings of a previous study evaluating the effect of UJ resection on cervical stability [20]. Analyzing the experimental results of stress distribution at the interface between the cervical endplate and bone implant will better our understanding of the potential mechanism of and factors associated with cage subsidence, which will enable surgeons to reduce the occurrence of cage subsidence in clinical practice [27].

The maximum contact pressure of the titanium plate and screw did not change significantly as the amount of UJ resection was increased in the states of flexion and backward extension, but increased significantly as the amount of UJ resection was increased in the states of lateral flexion and rotation. During flexion and extension, there was only a small change in the contact pressure of the titanium plate and screw, indicating that the interbody fusion cage plays a greater role in mechanical load sharing than the titanium plate and screw [28]. The contact pressure of the titanium plate and screw increased uniformly as the amount of UJ resection was increased, but the pressure was less in ACDF with unilateral UJ resection than ACDF with bilateral resection, indicating that unilateral UJ resection achieves good cervical spinal stability and that both unilateral and bilateral UJ resection do not result in stress concentration. These findings are similar to those of a previous study [29]. Therefore, we recommend reducing the amount of UJ resection and avoiding bilateral UJ resection as much as possible during anterior cervical surgery.

The maximum displacement of the cervical vertebrae refers to the displacement of two adjacent vertebral bodies, which reflects the relative stability of ACDF combined with different amounts of UJ resection [30]. Our results showed that although the interbody fusion cage, titanium plate, and screw are fixed during the operation, there is still a large maximum displacement of the cervical vertebrae in the flexion state, which may be one of the factors leading to subsidence of the interbody fusion cage after anterior cervical surgery. In addition, the maximum displacement of the cervical vertebrae did not change significantly with the increase in the amount of UJ resection in the states of flexion and extension. In the states of lateral flexion and rotation, the maximum displacement of the cervical vertebral bodies increased significantly as the amount of UJ resection increased, and this increase was more obvious in bilateral UJ resection than unilateral resection, especially in lateral flexion. These results show that the UJs mainly limit cervical lateral flexion, suggesting that excessive resection of the UJs and inappropriate postoperative neck brace application may lead to loosening of the internal fixation.

The spine is composed of complex and interconnected structures, including vertebral bodies, disks, facet joints, ligaments, and musculature, which together contribute to the stability and transmission of forces in the spine [31, 32]. These structures maintain the normal function of the spine, and the degeneration or injury of one structure affects the rest of the structures. In the present study, the standard model was based on a healthy cervical spine rather than a degenerated cervical spine, which may prevent the finite element analysis results from accurately representing postoperative outcomes of degenerated spines [33]. Compared with the healthy spine, the degenerated spine has decreased segmental motion, intervertebral space heights, and paravertebral muscle strength, and increased facet joint loading. Although the postoperative models were identical in structural composition, there were still large differences between models in the calculated parameters including range of motion, contact pressure, and disk pressure. In addition, the morphology of the pathological cervical spine may include variations in cervical curvature, loss of intervertebral disk height, and UJ hyperplasia, which leads to variations in the postoperative biomechanical results. To ensure the uniformity of the experiment, we used the CT data of a healthy young man. However, in future experiments, we need to establish a pathological degenerative spine model for biomechanical testing.

Our study has several limitations. The standard model was validated using an indirect method of comparison with published data [34, 35]. Although finite element analysis is an effective validation method, the accuracy of the results is reduced because of unclear in vitro experimental conditions, large standard deviations, and a lack of specific material properties in previous studies. Furthermore, as the current finite element method lacks a recognized construction method for the UJs, the UJs were simplified in the process of building the model, which may lead to the loss of some details and the deviation of the experimental results. Although the load application in the finite element test partially represents the effect of muscle on the cervical spine, the detailed and complex function of muscle on spinal movement cannot be simulated. Finally, although the CT data of a healthy man were used, there may be differences in bone size and muscle volume in accordance with different regions, ethnic groups, religions, and dietary habits, which may impact the results of finite element analysis. Overall, although our predictions may not represent accurate clinical values, they can predict the trends of changes in the cervical spine during different movements after ACDF combined with different amounts of UJ resection.

## Conclusions

On the basis of the stress distribution and vertebral body displacement in each state of the models in our study, we suggest that clinicians should reduce the UJ damage as much as possible while ensuring clinical efficacy when performing anterior cervical surgery. For CSR caused by hyperplasia of the uncinate joint, we suggest that the UJs should be resected by less than 40%. During the rehabilitation process after anterior cervical spine surgery, we recommend that patients wear a neck brace to limit flexion, lateral bending, and rotational movement to avoid subsidence of the cage and failure of internal fixation.

## Abbreviations

CSR: Cervical spine radiculopathy
ACDF: Anterior cervical decompression and fusion
UJ: Uncovertebral joint
3D: Three-dimensional
CT: Computed tomography
MRI: Magnetic resonance imaging
FLE: Flexion
EXT: Extension
LLB: Left lateral bending
RLB: Right lateral bending
LAR: Left axial rotation
RAR: Right axial rotation
